# Anxiety is associated with diminished exercise performance and quality of life in severe emphysema: a cross-sectional study

**DOI:** 10.1186/1465-9921-11-29

**Published:** 2010-03-09

**Authors:** Nicholas D Giardino, Jeffrey L Curtis, Adin-Cristian Andrei, Vincent S Fan, Joshua O Benditt, Mark Lyubkin, Keith Naunheim, Gerard Criner, Barry Make, Robert A Wise, Susan K Murray, Alfred P Fishman, Frank C Sciurba, Israel Liberzon, Fernando J Martinez

**Affiliations:** 1 Mental Health Service, VA Ann Arbor Healthcare System, Ann Arbor, MI, USA; 2 Division of Pulmonary and Critical Care Medicine, Department of Internal Medicine, University of Michigan Health System, Ann Arbor, MI, USA; 3 Department of Psychiatry, University of Michigan, Ann Arbor, MI, USA; 4 Pulmonary and Critical Care Medicine Section, VA Ann Arbor Healthcare System, Ann Arbor, MI, USA; 5 Division of Pulmonary & Critical Care Medicine, University of Washington, Seattle, WA, USA; 6 Department of Surgery, St Louis University, St Louis, MO, USA; 7 Division of Pulmonary & Critical Care Medicine, Temple University, Philadelphia, PA, USA; 8 Division of Pulmonary Sciences and Critical Care Medicine, National Jewish Medical Center and Research Center, University of Colorado, Denver, CO, USA; 9 Division of Pulmonary & Critical Care Medicine, Johns Hopkins University, Baltimore, MD, USA; 10 Department of Medicine, University of Pennsylvania, Philadelphia, PA, USA; 11 Division of Pulmonary & Critical Care Medicine, University of Pittsburgh, Pittsburgh, PA, USA

## Abstract

**Background:**

Anxiety in patients with chronic obstructive pulmonary disease (COPD) is associated with self-reported disability. The purpose of this study is to determine whether there is an association between anxiety and functional measures, quality of life and dyspnea.

**Methods:**

Data from 1828 patients with moderate to severe emphysema enrolled in the National Emphysema Treatment Trial (NETT), collected prior to rehabilitation and randomization, were used in linear regression models to test the association between anxiety symptoms, measured by the Spielberger State Trait Anxiety Inventory (STAI) and: (a) six-minute walk distance test (6 MWD), (b) cycle ergometry peak workload, (c) St. Georges Respiratory Questionnaire (SRGQ), and (d) UCSD Shortness of Breath Questionnaire (SOBQ), after controlling for potential confounders including age, gender, FEV_1 _(% predicted), DL_CO _(% predicted), and the Beck Depression Inventory (BDI).

**Results:**

Anxiety was significantly associated with worse functional capacity [6 MWD (B = -0.944, p < .001), ergometry peak workload (B = -.087, p = .04)], quality of life (B = .172, p < .001) and shortness of breath (B = .180, p < .001). Regression coefficients show that a 10 point increase in anxiety score is associated with a mean decrease in 6 MWD of 9 meters, a 1 Watt decrease in peak exercise workload, and an increase of almost 2 points on both the SGRQ and SOBQ.

**Conclusion:**

In clinically stable patients with moderate to severe emphysema, anxiety is associated with worse exercise performance, quality of life and shortness of breath, after accounting for the influence of demographic and physiologic factors known to affect these outcomes.

**Trail Registration:**

ClinicalTrials.gov NCT00000606

## Background

Chronic obstructive pulmonary disease (COPD) is a leading cause of disability and death. Disability, functional limitations and decreased quality of life in patients with COPD are correlated with objective physiologic measures of disease severity [1-3]. However, a large proportion of the variance in functional status and quality of life associated with COPD is not explained by measures of pulmonary physiology. Psychological factors may play in important role in determining the impact of COPD on patient functioning. For example, anxiety in patients with COPD has been associated with decreased quality of life, more severe dyspnea, greater disability, and impaired functional status [4,5] even after controlling for lung function and the presence of other chronic diseases [6,7]. Anxiety is also a significant predictor of the frequency of hospitalizations for acute exacerbations of COPD [8].

Anxiety is a major clinical problem in patients with COPD. The prevalence of clinical anxiety disorders in patients with COPD is substantially higher than in the general public. Anxiety symptoms are very common in patients with COPD. In previously published studies 10-80% of patients endorsed anxiety symptoms [1,7,9-12], exceeding that for patients with other chronic medical conditions such as heart disease, renal disease, AIDS and cancer [13,14]. Estimates for specific anxiety *disorders *range from a 10-32%, a 3- to 10-fold increase in COPD compared to the general population. The most common anxiety disorders diagnosed with COPD are generalized anxiety disorder and panic disorder, which may occur in as many as one-third of COPD patients [15-17]. Defining the contribution of anxiety to functional impairment in COPD is a first step in determining the potential for interventions to combat anxiety to improve functional status.

Most previous studies of the impact of anxiety on patients with COPD have relied on patient self-report measures of functioning [4,6,7]. In these studies self-report biases may confound the true association between psychological and physical health [18]. Investigations that have included objective measures of functioning (e.g., 6-minute walk distance test (6 MWD)) have been limited by small sample size, or have included only patients selected for high levels of anxiety and depression [5,9].

In the current study, we hypothesized that anxiety would be associated with worse functional performance (6 MWD; maximal exercise capacity), health-related quality of life (SGRQ), and dyspnea (SOBQ), after controlling for the effects of potential confounders including age, pulmonary function and depression. We used data from a carefully characterized large group of patients with severe emphysema who were evaluated for the National Emphysema Treatment Trial (NETT). We also examined whether sex differences existed in the relationship between anxiety symptoms and outcomes variables (6 MWD, maximal exercise capacity, SGRQ, SOBQ).

## Methods

### Study group

Ethics committee approval for the NETT was obtained from the Institutional Review Boards of all participating sites. Procedures for recruitment, screening, determination of eligibility, and assessments for the NETT are described in detail elsewhere [19,20]. Briefly, 3777 patients were screened for the NETT from 1998 to 2002. Patients were included if they had moderate to severe emphysema, had been nonsmokers for at least 6 months, and did not have clinically significant comorbid conditions or circumstances that placed them at high risk for perioperative morbidity or mortality or made it unlikely that they would complete the trial. In order to maximize generalizability and sample heterogeneity, patients were included in the analyses if they had all of the required data from the initial screening assessment, prior to the start of the pre-randomization rehabilitation program. 1828 patients met these criteria. A total of 1218 patients went on to randomization to receive either lung volume reduction surgery or continued regular medical treatment. Patients failed to reach randomization for a number of reasons, for example failure to complete rehabilitation program and all postrehabilitation and randomization assessments, failure to obtain final approval for surgery just prior to randomization, and other new onset complications that met study exclusion criteria. Although no differences between randomized and non-randomized patients were expected in our analyses, in order to allow comparison with a number of other reports that are based on data only from randomized NETT subjects, our data analyses were also repeated for randomized patients with complete data from the initial screening assessment on all variables analyzed (*n *= 1154).

### Demographic and questionnaire measures

Demographics and self-report measures were collected using standardized instruments including:

#### Spielberger State Trait Anxiety Inventory (STAI; [21])

The STAI is a 40-item self-report measure of enduring (trait) and transient (state) anxiety symptoms. Respondents rate how statements reflect how they generally feel on a 4-point scale. STAI state and trait scores range from 20-80. State anxiety is defined as unpleasant emotional arousal, characterized by feelings of tension and apprehension, and heightened autonomic nervous system activity, while trait anxiety measures a stable tendency to respond with state anxiety. The state anxiety scale of the questionnaire was chosen for our analyses in order to more closely match the time frame referenced by most of our other questionnaire measures (i.e., current, versus past or typical, functioning). In addition the state anxiety measurement has been shown to be more valid than that of trait anxiety [22].

#### Beck Depression Inventory (BDI;[23])

The BDI is a 21 item self-report measure of symptoms of depression. Respondents choose statements that reflect how they have felt over the past 2 weeks. The BDI contains 13 items assessing cognitive-mood depressive symptoms (e.g., sadness, guilt) and 8 items that assess physical-performance symptoms of depression (e.g., fatigue, weight loss, physical health worries).

#### St. Georges Respiratory Questionnaire (SGRQ;[24])

The SGRQ is a 60-item-disease specific instrument developed for use with patients with airflow limitation and designed to measure health-related quality of life. We used the full-scale SGRQ score in our analysis.

#### UCSD Shortness of Breath Questionnaire (UCSD-SOBQ[25])

The UCSD-SOBQ is a 33-item measure of shortness of breath while performing activities.

Questionnaires were administered to subjects after performance of diagnostic testing and determination of study eligibility.

### Physiologic measures

Pulmonary function tests, including spirometry and single-breath diffusing capacity (DL_CO_), were performed in accordance with American Thoracic Society standards[26,27]. Spirometry values used in this report (FEV_1_) were obtained following bronchodilator (albuterol) administration. Percent of predicted values were calculated using normal reference values derived from Crapo and colleagues [28,29]. The standardized 6 MWD protocol has been described in detail elsewhere [30] and provided maximum distance walked. Maximum exercise capacity (watts) was measured on an electromagnetically-braked cycle ergometer that increased at a rate of 5 or 10 W per minute after 3 minutes of unloaded pedaling while subjects breathed 30 percent oxygen.

### Statistical analysis

Descriptive statistics were performed. Analysis of variance was used to compare patients who were versus were not randomized in the NETT. For categorical variables a Pearson Chi-square test was performed. Next, four separate multiple linear regression models were computed to test the association between state anxiety and: (a) 6 MWD; (b) maximum exercise capacity; (c) St. Georges Respiratory Questionnaire total score; and (d) UCSD Shortness of Breath Questionnaire total score. In the first regression model, 6 MWD was entered as the dependent variable. Patient age, gender, FEV_1_%, DL_CO_%, Beck Depression Inventory score and Spielberger State Anxiety score were entered as the independent variables. The second through fourth models were identical to the first, except substituting maximum workload on cardiopulmonary exercise test, SGRQ total score, and UCSD SOBQ total score, respectively, as the dependent variable. In order to test for possible collinearity between independent variables, eigenvalues of the scaled and uncentered cross-products matrix, condition indices, variance-decomposition proportions, variance inflation factors (VIF) and tolerances were computed for individual variables. All statistical analyses were performed using SPSS statistical software (SPSS, Inc. Chicago, IL, USA). A probability value of p = .05 was used to determine statistical significance.

The STAI and BDI are not clinical diagnostic tools. However there are published cutoff scores for both instruments to indicate clinically significant symptom levels. In general population samples, cutoff scores of 19 and 40 are used for the STAI [21] and BDI [23], respectively, to indicate clinically significant symptoms and likely diagnosis. For the BDI, scores of 10 or above indicate mild-moderate depressive symptoms. In geriatric outpatient populations higher cutoff scores have been recommended: 22 for the STAI and 44 for the BDI, based on assessment studies in persons aged 55 and older [31,32]. Questions have also been raised about the interpretation of specific depression symptoms in patients with chronic medical illness, including COPD [33,34]. Because of a concern that the full BDI score might overestimate depression severity in patients with more severe emphysema due to overlap between COPD severity and somatically-focused depression symptoms on the BDI (e.g., fatigue, weight loss, physical health worries), we repeated each multiple regression analysis entering the totals of 'cognitive-mood' and 'physical-performance' BDI items separately as independent variables.

## Results

In general, this study population was elderly and primarily white; approximately two-thirds were male. Subjects' age range was 28-89 years, with 96% of patients aged 55 or older. Subjects had severe airflow limitation and impaired diffusing capacity. Compared to published population norms for the SGRQ (mean = 12.17) [35] and 6 MWD (mean = 555 meters)[36] in similar age groups (ages 60-69), subjects in this study showed lower exercise performance and poorer health-related quality of life (Table 1).

**Table 1:** Characteristics of enrolled NETT patients used in this report* (n = 1828).

Age at evaluation (years; Mean ± SD)	66.7 ± 6.3
	
Gender	1134 (62%) Male
	694 (38%) Female
	
Race/ethnic group	1727 (94%) Non-Hispanic white
	73 (4%) Non-Hispanic black
	28 (2%) Other
	
FEV_1_	
% of predicted (Mean ± SD)	27.1 ± 7.5
L (Mean ± SD)	0.78 ± 0.25
	
D_L_CO	
% of predicted (Mean ± SD)	28.2 ± 10.1
ml/min/mmHg STPD (Mean ± SD)	8.0 ± 3.2
	
Beck Depression Inventory (Mean ± SD)	9.5 ± 6.0
	
Spielberger Anxiety Inventory - State (Mean ± SD)	34.9 ± 10.9
	
6-Minute Walk Test (meters; Mean ± SD)	344.2 ± 105.8
	
Maximum Exercise Capacity (watts; Mean ± SD)	35.6 ± 21.9
	
St. George's Respiratory Questionnaire - total score (Mean ± SD)	56.4 ± 13.1
	
UCSD Shortness of Breath Questionnaire (Mean ± SD)	64.9 ± 19.4

* Includes some patients who were not ultimately randomized to treatment. See Methods for additional details

Subjects showed moderately high levels of baseline anxiety and depression. Thirty percent of subjects had state anxiety scores above 40 and twenty percent scored above 44. Forty-one percent of subjects had a BDI score of 10 or higher, indicating mild-moderate depressive symptoms. Eight percent of subjects scored 19 or higher on the BDI and 4% scored 22 or higher, indicating moderate-severe symptoms. Women had significantly higher anxiety (36.4 vs. 34.0, p < .001) and depression (10.3 vs. 9.0, p < .001) scores than men. Non-randomized subjects differed significantly from randomized subjects in 6 MWD (337.3 m. vs. 348.0 m., p = .04) and shortness of breath scores (63.5 vs. 65.5, p = .04), with non-randomized subjects showing greater impairment on both variables.

Results of multiple linear regression analyses showed that anxiety was significantly associated with decreased 6 MWD, even after adjusting for patient age, gender, FEV_1 _(% predicted), DL_CO _(% predicted), and depression (Table 2). Likewise, anxiety was significantly and inversely associated with maximal workload during cardiopulmonary exercise testing after adjusting for age, gender, FEV_1 _%, DL_CO _%, and depression. Finally, after controlling for patient age, gender, FEV_1 _%, DL_CO_%, and depression score, anxiety was also significantly associated with SGRQ and UCSD Shortness of Breath Questionnaire total scores. Regression coefficients from models with the BDI mood and physical symptom scores show that a 10-point increase in anxiety score is associated with a mean decrease in 6 MWD of 9 meters, a decrease in maximum exercise workload of almost 1 Watt, and an increase of approximately 2 points on the SGRQ and the UCSD SOBQ. Collinearity diagnostic test did not indicate significant collinearity between independent variables in the regression models (data not shown). Effects for anxiety were similar when limiting analyses to only randomized patients, but were not statistically significant for maximum workload (data not shown).

**Table 2:** Results of multiple linear regression analyses for 6 MWD, maximum exercise, quality of life, and shortness of breath.

						Model fit	
	**B**	**SE**	**Beta**	***p***	**95% Confidence Interval for B**	**R^2^**	***p***

**6MWD**							
Age (years)	-2.37	.37	-.14	<.001	[-3.09, -1.65]		
Male Gender	45.04	4.78	.21	<.001	[35.67, 54.40]		
FEV_1_%	3.39	.33	.25	<.001	[2.75, 4.03]		
DL_CO_%	2.61	.23	.25	<.001	[2.15, 3.06]		
Depression-M	.26	.79	.01	.746	[-1.30, 1.81]		
Depression-P	-2.64	.85	-.08	.002	[-4.31, -.97]		
Anxiety	-.99	.24	-.10	<.001	[-1.45, -.52]	.22	<.001
							
**Max. exercise**							
Age (years)	-.62	.07	-.18	<.001	[-.75, -.49]		
Male Gender	20.44	.85	.45	<.001	[18.77, 22.11]		
FEV_1_%	1.14	.06	.40	<.001	[1.03, 1.26]		
DL_CO_%	.50	.04	.23	<.001	[.42, .58]		
Depression-M	.21	.14	.03	.142	[-.07, .49]		
Depression-P	-.73	.15	-.11	<.001	[-1.02, -.43]		
Anxiety	-.10	.04	-.05	.017	[-.19, -.02]	.43	<.001
							
**Quality of life**							
Age (years)	-.26	.04	-.13	<.001	[-.35, -.18]		
Male Gender	1.18	.56	.04	.037	[.07, 2.29]		
FEV_1_%	-.10	.04	-.06	.012	[-.17, -.02]		
DL_CO_%	-.07	.03	-.05	.018	[.-12, -.01]		
Depression-M	.31	.09	.09	.001	[.12, .49]		
Depression-P	1.34	.10	.33	<.001	[1.15, 1.54]		
Anxiety	.187	.03	.16	<.001	[.13, .24]	.28	<.001
							
**Shortness of Breath**							
Age (years)	-.132	.07	-.04	.045	[-.26, -.01]		
Male Gender	-2.940	.85	-.07	.001	[-4.61, -1.27]		
FEV_1_%	-.345	.06	-.14	<.001	[-.46, -.23]		
DL_CO_%	-.285	.04	-.15	<.001	[-.37, -.20]		
Depression-M	.139	.14	.03	.331	[-.14, .42]		
Depression-P	1.836	.15	.30	<.001	[1.54, 2.13]		
Anxiety	.206	.04	.12	<.001	[.12, .29]	.24	<.001

Unstandardized regression coefficients (B), standard errors (SE), standardized coefficients (Beta), significance values (*p*), and 95% confidence intervals are shown for each independent variable. Goodness-of-fit (R^2^) shown for each regression model. For each regression model, BDI depression was entered either as total score or as two separate independent variables composed of the mood (Depression-M) or physical (Depression-P) BDI symptom items.

FEV_1_%: forced expiratory volume in 1 second, percent of predicted.

DL_CO_%: single-breath diffusing capacity of carbon monoxide, percent of predicted.

Total BDI depression score was also significantly associated with 6 MWD (B = -1.12, SE = 0.43, p = .009), peak workload (B = -0.24, SE = .08, p = .002), SGRQ (B = .80, SE = 0.05, p < .001) and UCSD Shortness of Breath Questionnaire (B = 0.950, SE = 0.08, p < .001) total scores. But, when BDI scores were separated into 'mood' and 'physical' symptoms scores, physical, but not mood, symptoms were associated with the dependent variables in all cases (Table 2).

In separate multivariate regression models predicting 6 MWD, maximum exercise workload, SGRQ, and UCSD Shortness of Breath Questionnaire total scores, a significant interaction was found between gender and anxiety score in predicting SGRQ total score after adjusting for age, FEV1%, DLCO%, and depression score. Anxiety was much more strongly associated with SGRQ for men (B ± SE = 0.23 ± .04; p < .001; 95% confidence interval [0.16, 0.30]), than for women (B ± SE = 0.09 ± .04; p = .03; 95% confidence interval [0.01, 0.18].

## Discussion

This analysis of a large, prospectively studied cohort of patients with severe emphysema screened for enrollment in a clinical trial of lung volume reduction surgery makes several important observations about state anxiety. We show that state anxiety was significantly and independently associated with 1) shorter 6 MWD distance; 2) diminished maximum workload on cardiopulmonary exercise testing; 3) poorer health-related quality of life, and 4) more shortness of breath. It should be emphasized that our measure of anxiety evaluates a general state of feeling anxious and does not indicate how anxious patients actually felt during exercise testing. Emotional distress experienced during exercise may be even more strongly associated with performance outcomes [37-39]. Nonetheless, our findings suggest that anxiety may be a valid target for therapeutic interventions in patients with severe emphysema.

A key feature of our report is the objective measurement of physical functioning using well-validated physiologic measures, which allows us to extend the findings of other investigators who suggested greater impairment in physical functioning and quality of life with increasing levels of anxiety in COPD patients [7,40-42]. Our results contrast with those of Borak et al., who reported no significant effect of anxiety on exercise performance [9], however the differences may relate mostly to methodological and data analytical issues. That study examined the effects of a number of psychological variables, including anxiety and depression, on 6 MWD in a group of 49 patients with moderate to severe COPD, and concluded that they had no effect at all on exercise performance. In their analyses, authors entered up to 15 independent variables into a multiple regression equation, with 6 MWD as the dependent variable. With only 49 patients, this model may have been underpowered to detect anything but very large effects. In addition, the power to detect an effect of anxiety was decreased further by the conversion of anxiety to a categorical variable of low, moderate or high based on subjects' scores on a continuous measure of anxiety. In contrast, our study analyzed anxiety as a continuous variable and, to our knowledge, utilized the largest sample size to date to examine the question.

Our results also contrast with those of Weaver and colleagues [43], who tested a causal model of factors affecting self-reported physical, mental, and social functioning including age, length of illness, FEV_1_, dyspnea, depression, anxiety, self-esteem, and exercise capacity, as measured by the 12-minute walk test in patients with COPD. They found that anxiety was linked to exercise capacity, but only through its association with depression and dyspnea. It is possible that the differences in findings between these studies are due to the use of different measures of anxiety, depression, and dyspnea. In addition, our subjects were a more homogenous group with more severe COPD and an emphysematous phenotype.

Our analyses showed that depression, as measured by the BDI total score, was significantly associated with poorer exercise performance and worse health-related quality of life scores. But, our results indicate that the observed association between depression and other patient variables was due to the somatic symptoms of depression included on the BDI. These include, for example, "I get tired more easily than I used to", "It takes an extra effort to get started at doing something", and "My appetite is not as good as it used to be." It is easy to see that these depression symptoms are also likely to be indicators of COPD severity. Thus, it is difficult to conclude that the observed associations between BDI total score and patient functioning were related to depression *per se*, rather than items on the BDI that served as another proxy for COPD severity. Future studies should use measures of depression that minimize overlap with COPD symptoms.

Our analysis of the effect of gender on the association between anxiety and functioning found an interaction between gender and anxiety on quality of life reports. For men, higher anxiety was associated worse health-related quality of life, as measured by the SGRQ total score. For female patients this association was much weaker, although still statistically significant. This finding is somewhat surprising given that greater emotional distress and lower health satisfaction and quality of life have been found for women with COPD in many [44-47], but not all [48,49] studies. In our data also, women reported greater symptoms of anxiety and depression than did men. Nonetheless our finding suggests that anxiety may impact men's ratings of health-related quality of life more than for woman. This interaction effect was not found for exercise performance or shortness of breath, indicating that the gender difference in the association between anxiety and quality of life is not due to a greater adverse impact of dyspnea or impaired physical functioning on quality of life for men. However it may be that anxiety has a greater impact on important activities and roles in men with COPD. Future research could be designed to more specifically study this potentially important phenomenon.

Several mechanisms, not mutually exclusive, may explain the link between anxiety and functional impairment in patients with COPD. First, anxiety may increase disability in COPD by increasing vigilance for, and amplification of, distressing respiratory sensations. The tendency to misinterpret ambiguous or potentially threatening stimuli, a characteristic of many anxiety disorders, would lead anxious COPD patients to avoid any activity that might produce these sensations. Second, patients with higher anxiety may be more emotionally sensitive to unpleasant somatic sensations, which would to lead to greater distress when these bodily cues are encountered. In a recent population-based longitudinal study, anxiety and depression were associated with the new onset of dyspnea, but not vice versa [50]. Third, longitudinal experience with COPD symptoms may generate fearful or catastrophic beliefs about respiratory sensations, which, in turn, provoke anxiety that limits engagement in physical activity. Patients with COPD and panic disorder report more fearful thoughts about, and avoidance of, unpleasant somatic sensations than COPD patients without anxiety [51]. Patients who endorse beliefs such as "dyspnea is always a sign of danger", or "activities that produce dyspnea make my COPD worse and should be avoided" are more functionally impaired and report poorer quality of life independent of COPD severity [52]. Near-death episodes, need for ventilatory support, and other illness experiences could also influence the development of these fearful beliefs and frightening thoughts.

Why anxiety is so much more common in COPD than in the general population or in other disease states remains unclear. Repetitive experiences with hypoxia and hypercapnia might sensitize neural circuits that control fear responses, such as neurons in the amygdala and locus ceruleus, to overreact to either subsequent episodes of hypoxia and hypercapnia or to fearful perceptions of conditioned stimuli such as the sensation of breathlessness [53]. These reactions would again lead to avoidance of physical activity and limit exercise performance. In addition, a vicious circle may ensue, in which dyspnea leads to anxiety, which produces a rapid, shallow breathing pattern, leading to air trapping and hyperinflation, creating further dyspnea and exercise limitations [54]. Thus, anxiety in the context of COPD may represent a 'normal' response to the anxiogenic experience of repeated dyspnea, hypoxia, or hypercapnia; or it may reflect the presence of a pre-morbid anxiety problem that may become exacerbated in the presence of COPD symptoms. Deciphering the relative contributions of these two pathways to anxiety will require longitudinal studies beginning in more mild stages of COPD.

Our findings suggest that screening for anxiety may be important in patients with moderate to severe COPD. Treating anxiety when present in patients with COPD may not only reduce emotional distress, but also improve physical functioning and overall quality of life. Brief screening instruments have been validated for the detection of anxiety in medical settings [55]. And, while there are no published large randomized controlled studies of treatments for anxiety disorders in patients with COPD, number of studies report decreased anxiety symptoms in COPD with antidepressant therapy [56-59], cognitive-behavioral therapy [60,61], or exercise therapy, including pulmonary rehabilitation [11,62-66].

Our findings from our analysis of data from the NETT have some limitations. As with all research reports from treatment trials such as the NETT, the most serious limitation is related to subject characteristics influenced by study selection criteria and subject self- selection. Subjects in this study had moderate to severe emphysema. In addition, subjects who participated in the NETT were not current cigarette smokers and had agreed to participate in a rigorous pulmonary rehabilitation program. As a result, it is important to note that our findings may not be generalizable to all patients with COPD. Likewise, since only patients with COPD were included (i.e., there was not a non-COPD control group), our results are not generalizable to other patient groups. Second, questionnaires were administered prior to pulmonary rehabilitation and treatment randomization. Thus, while the measures were thus not influenced by the impact of rehabilitation or treatment, it is possible that subjects may have experienced heightened anxiety or mood symptoms in anticipation of rehabilitation participation or treatment assignment.

While the associations between state anxiety and exercise performance found in our study were statistically significant, the absolute effect sizes for anxiety were modest. We found that a 10-point increase in state anxiety score is associated with a mean decrease in 6 MWD of 9 meters, a decrease in maximum exercise workload of approximately 1 Watt, and an increase of approximately 2 points on the SGRQ and the UCSD SOBQ. Published guidelines for the 6 MWD suggest 50 meters change to indicate clinically significant changes in walk distance [67]. For the SGRQ a change in 4 points is used to indicate clinically important differences in health-related quality of life [24]. However these guidelines were based on within-subject changes observed in clinical trials, rather than between-subject differences in cross-sectional studies. Nonetheless, using these figures as a rough guide, a patient with a high state anxiety score (2 SD above the mean or STAI = 57) in our sample would be expected to walk about 40 meters less on the 6 MWD and score 9 points worse on the SGRQ than a patient with low state anxiety (2 SD below the mean, or STAI score = 13). Thus, while the effect of anxiety on patients with COPD appears to be clinically meaningful, the significance of our findings needs to be further evaluated.

## Conclusion

In summary, we found that state anxiety is associated with worse functioning on measures of exercise performance, health-related quality of life and shortness of breath in patients with moderate to severe emphysema, after accounting for the influence of demographic and physiologic factors known to affect these outcomes. Our results support the need for additional research into the role of anxiety as an important source of functional impairment and decreased quality of life in patients with COPD. Future studies will need to examine the mechanisms by which anxiety impacts exercise performance.

## Competing interests

The authors declare that they have no competing interests.

## Authors' contributions

NDG drafted the manuscript and performed statistical analyses. JLC, JOB, KN, GC, BM, RAW, APF, FCS, and FLM participated in the design and coordination of the study, interpretation of data and writing the manuscript. AA and SKM participated in statistical analyses and writing the manuscript, VSF, ML and IL participated in writing the manuscript. All authors read and approved the final manuscript.
